# Emerging Infectious Diseases: a 10-Year Perspective from the National Institute of Allergy and Infectious Diseases

**DOI:** 10.3201/eid1104.041167

**Published:** 2005-04

**Authors:** Anthony S. Fauci, Nancy A. Touchette, Gregory K. Folkers

**Affiliations:** * National Institutes of Health, Bethesda, Maryland, USA

**Keywords:** emerging diseases, HIV, tuberculosis, malaria, SARS, West Nile virus, biodefense, vaccines, pandemic, influenza, perspective

## Abstract

Although optimists once imagined that serious infectious disease threats would by now be conquered, newly emerging (e.g., severe acute respiratory syndrome [SARS]), reemerging (e.g., West Nile virus), and even deliberately disseminated infectious diseases (e.g., anthrax bioterrorism) continue to appear throughout the world. Over the past decade, the global effort to identify and characterize infectious agents, decipher the underlying pathways by which they cause disease, and develop preventive measures and treatments for many of the world's most dangerous pathogens has resulted in considerable progress. Intramural and extramural investigators supported by the National Institute of Allergy and Infectious Diseases (NIAID) have contributed substantially to this effort. This overview highlights selected NIAID-sponsored research advances over the past decade, with a focus on progress in combating HIV/AIDS, malaria, tuberculosis, influenza, SARS, West Nile virus, and potential bioterror agents. Many basic research discoveries have been translated into novel diagnostics, antiviral and antimicrobial compounds, and vaccines, often with extraordinary speed.

## Abstract 2

Advances in infectious disease research over the past 10 years have allowed breakthroughs in the

diagnosis, prevention, and treatment of infectious disease.

Infectious diseases have been an ever-present threat to mankind. From the biblical plagues and the Plague of Athens in ancient times, to the Black Death of the Middle Ages, the 1918 "Spanish Flu" pandemic, and more recently, the HIV/AIDS pandemic, infectious diseases have continued to emerge and reemerge in a manner that defies accurate predictions (*1**–**3*).

The past 10 years (1994–2004) have been no exception, as many new and reemerging microbial threats have continued to challenge the public health and infectious disease research communities worldwide. Since 1994, when Emerging Infectious Diseases made its publication debut, significant strides in the global fight against the HIV/AIDS pandemic have been made. The infectious disease community has confronted several other newly emerging pathogens, such as the severe acute respiratory syndrome–associated coronavirus (SARS-CoV), henipaviruses (Hendra and Nipah), and, most recently, avian influenza viruses that have caused illness and deaths in humans with the threat of evolution into a pandemic (*1**–**3*). In addition, historically established infectious diseases, such as West Nile fever, human monkeypox, dengue, tuberculosis, and malaria have reemerged or resurged, sometimes in populations that previously had been relatively exempt from such affronts. Over the past decade, strains of common microbes such as *Staphylococcus aureus* and *Mycobacterium tuberculosis* have continued to develop resistance to the drugs that once were effective against them (*1**–**4*). Such antimicrobial-resistant microorganisms, which defy conventional therapies and pose a threat to public health, underscore the need for a robust pipeline of new antimicrobial agents based on innovative therapeutic strategies, new vaccines, and other preventive measures (*3**,**4*).

Perhaps most disturbing, the United States has recently experienced a deliberately spread infectious disease in the form of 22 anthrax infections, including 5 anthrax-related deaths resulting from bioterrorism in 2001 (*5*). These cases were accompanied by widespread psychological sequelae and societal and economic disruptions.

These emerging and reemerging infectious diseases are superimposed on a substantial baseline of established infectious diseases. Although annual deaths and lost years of healthy life from infectious diseases have decreased over the past decade, the worldwide impact from infectious diseases remains substantial. Overall, infectious diseases remain the third leading cause of death in the United States each year and the second leading cause of death worldwide (*6*). As shown in Figure 1, of the estimated 57 million deaths that occur throughout the world each year, ≈15 million, >25%, are directly caused by infectious diseases. Millions more deaths are due to secondary effects of infections (*6*).

**Figure 1:**
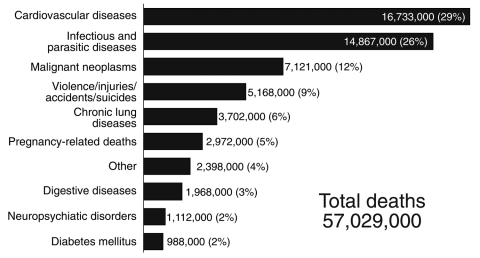
Leading causes of death worldwide (estimates for 2002). Nearly 15 million (>25%) of the 57 million annual deaths worldwide are caused by infectious disease (*6*).

Infectious diseases also lead to compromised health and disability, accounting for nearly 30% of all disability-adjusted life years (DALYs) worldwide (1 disability-adjusted life year is 1 lost year of healthy life) (*6*). Infectious diseases that contribute to the nearly 1.5 billion total DALYs each year are categorized in Figure 2.

**Figure 2:**
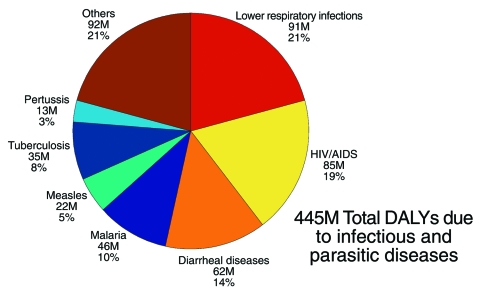
Leading causes of disability-adjusted life years (DALYs) due to infectious and parasitic diseases (2002 estimates). Lower respiratory infections, HIV/AIDS, diarrheal diseases, and malaria are among the infectious diseases that contribute to the most DALYs each year throughout the world (*6*).

In the United States, the Centers for Disease Control and Prevention has devised strategies to prevent, monitor, and contain disease outbreaks. Within the National Institutes of Health, the National Institute of Allergy and Infectious Diseases (NIAID) is the lead agency for infectious disease research.

Over the past decade, the NIAID budget has quadrupled; spending on emerging infectious diseases has increased from <$50 million in 1994 to >$1.7 billion projected for 2005, a boost due in large part to increases in funding for biodefense research (Figure 3). NIAID-supported intramural and extramural investigators have contributed substantially to the global effort to identify and characterize infectious agents, decipher the underlying pathways by which they cause disease, and develop preventive measures and treatments for many of the world's most dangerous pathogens. This review briefly highlights some of the research strides made by NIAID-supported investigators during the past decade in preventing and combating emerging and reemerging infectious diseases threats.

**Figure 3:**
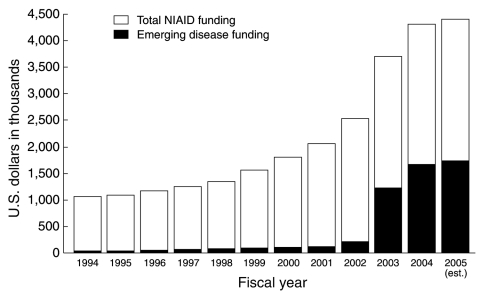
Budget of the National Institute for Allergy and Infectious Disease (NIAID), FY1994–2005. The overall NIAID budget rose from $1.06 billion in FY1994 to $4.4 billion (estimated) in FY2005. Funding for emerging infectious diseases rose from $47.2 million in FY1994 to $1.74 billion in FY2005 (est.).

## HIV/AIDS

HIV/AIDS has resulted in the death of >20 million persons throughout the world and is the leading cause of death among persons 15–59 years of age. Approximately 40 million persons are estimated to be living with HIV infection (*7*). In the United States, an estimated 1 million persons are infected with HIV, and 40,000 new infections occur each year. Since its recognition in 1981, the disease has killed more than half a million people in the United States (*8*).

Despite these grim statistics, reason for hope exists. Basic research has yielded major insights into the pathogenic mechanisms of HIV disease. This knowledge paved the way for the development of >20 antiretroviral medications approved by the Food and Drug Administration (FDA) that target HIV, as well as novel strategies for prevention and vaccine development (*9*).

**Figure 4:**
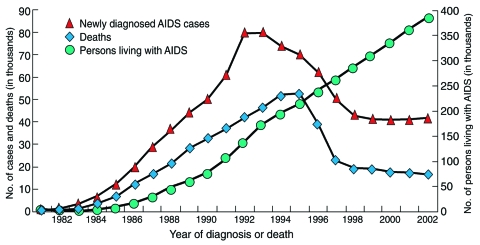
AIDS cases, AIDS deaths, and persons living with AIDS in the United States, 1981–2003. Over the past decade, the number of new AIDS cases and deaths due to AIDS has decreased, while the number of people living with the disease has increased, due in large part to improvements in diagnosis and treatment. Estimates adjusted for reporting delays. Source: CDC (*8*).

With the use of combinations of drugs that target different proteins involved in HIV pathogenesis (a treatment strategy known as highly active antiretroviral therapy [HAART]), rates of death and illness in the United States and other industrialized countries have been dramatically reduced (*7**,**8*) (Figure 4). Although the death rate due to HIV/AIDS in Europe and North America has fallen by 80% since HAART was introduced, relatively few people in poor countries have reaped these benefits. New initiatives such as the Global Fund to Fight AIDS, Tuberculosis, and Malaria and the President's Emergency Plan for AIDS Relief promise to greatly reduce the disparity between rich and poor countries with regard to access to HIV treatment, care, and prevention services (*7*).

The greatest challenge in HIV/AIDS research remains developing a vaccine that can either prevent the transmission of the virus or, failing that, halt progression to AIDS. Since 1987, NIAID has funded >70 clinical trials evaluating >50 different HIV vaccine candidates. Unfortunately, the first large-scale phase 3 trial of an HIV vaccine reported in 2003 had disappointing results (*10*). Many different vaccine strategies, including viral and bacterial vectors, DNA vaccines, viruslike particle vaccines, and peptide vaccines are being investigated, and ≈15 clinical trials in humans are under way. The effects of various adjuvants and different routes of administration also are being tested.

HIV vaccine developers face formidable scientific obstacles, including the virus's genetic diversity and the lack of a clear understanding of the correlates of protective immunity in HIV infection (*9*). A critical and so far elusive milestone is the discovery of a stable and immunogenic conformational epitope of the HIV envelope that would elicit broadly reactive neutralizing antibodies against primary isolates of HIV (*9*). To overcome these challenges, collaborations involving government, academia, industry, and philanthropies and new cross-sector partnerships such as the Global HIV Vaccine Enterprise, a virtual consortium of independent organizations, are being established to advance HIV vaccine research and foster greater collaboration among HIV vaccine researchers worldwide (*11*).

## Malaria

The social, economic, and human toll exacted by malaria globally is widespread and profound. Each year, acute malaria occurs in >300 million people and results in >1 million deaths worldwide. Most of these deaths occur in young children who live in sub-Saharan Africa (*3**,**6*).

In humans, the disease is caused by one of 4 species of *Plasmodium*, a single-cell parasite transmitted by anopheline mosquitoes. In 2002, the complete genomic sequence of *Plasmodium falciparum* as well as that of the mosquito vector *Anopheles gambiae* were completed as the result of a multinational effort (*12**,**13*). With the genomic sequences of the parasite and its human and mosquito hosts now available, researchers have powerful tools to further characterize the genes and proteins involved in the life cycle of the parasite, and they are using this information to design effective drugs and vaccines.

Drug-resistant *Plasmodium* strains are widespread, as are insecticide-resistant strains of the mosquitoes that carry the parasites. Mutations in both parasites and mosquitoes that confer drug and insecticide resistance have been identified. For example, genetic analysis and molecular epidemiology studies of *P. falciparum* have shown that resistance to chloroquine and other antimalarials is caused by a mutation in a single gene, called *pfcrt* (*14**,**15*). This information is being used to track the spread of drug-resistant strains of the parasite and identify new drug targets (*16*). Researchers also are exploiting the new genomic information to create genetically altered mosquitoes that resist parasite infection and to develop new compounds that overcome or avoid resistance to existing pesticides (*17*).

Developing an effective antimalarial vaccine has been a challenge; however, an international research team recently developed a vaccine that shows promise in preventing malaria among children in Mozambique. The vaccine prevented infection and severe disease in a substantial percentage of children tested, a breakthrough with the potential of saving millions of lives (*18*). Specifically, vaccine efficacy for the first clinical episodes was 29.9% (95% confidence interval [CI] 11.0–44.8, p = 0.004). Vaccine efficacy for severe malaria was 57.7% (95% CI 16.2–80.6, p = 0.019) (*18*). In addition, at least 35 other malaria vaccine candidates have undergone phase 1 clinical trials in humans, and 13 have moved into more advanced clinical development (*19*). Preclinical development of more than a dozen other candidates is being supported (*19*).

## Tuberculosis

Another ancient microbial scourge that has reemerged in recent years is tuberculosis (TB), caused by infection with the bacterium *Mycobacterium tuberculosis*. This infection is estimated to be prevalent in one third of the world's population. From this reservoir, 8 million new cases of TB develop worldwide each year that carry a death toll of >2 million (*3**,**6*). TB is especially prevalent among persons infected with HIV. The only currently available TB vaccine, *M. bovis* bacillus Calmette-Guérin (BCG), offers some protection, but its effect diminishes with time (*20*). TB drug treatment is effective, but adherence to lengthy therapeutic regimens is difficult to maintain, and multidrug-resistant TB is on the rise in many countries (*3**,**4*).

Researchers are applying state-of-the-art genomic and postgenomic techniques to identify key molecular pathways that could be exploited to develop improved TB interventions and vaccines (*21**,**22*). In 2004, for the first time in 60 years, 2 new vaccines designed to prevent TB entered phase 1 clinical trials in the United States (*23**,**24*). Many promising new anti-TB drug candidates also are now entering the drug pipeline (*25*). Derivatives of known anti-TB drugs, such as thiolactomycin and ethambutol, are currently being screened for activity against *M. tuberculosis*. Preclinical development of a highly promising candidate, SQ109 is nearing completion.

## Influenza

Each year, influenza develops in up to 20% of all Americans, and >200,000 are hospitalized with the disease. Although influenza is commonplace and generally self-limited, an estimated 36,000 Americans die each year from complications of the disease (*26*). Worldwide, severe influenza infections develop in 3–5 million people annually, and 250,000–500,000 deaths occur (*27*).

Outbreaks of avian influenza recently have drawn attention worldwide, particularly in Southeast Asia, where at least 55 persons have been infected and 42 have died since January 2004. The current strain of H5N1 avian influenza is highly pathogenic; it has killed millions of chickens and other birds. Although the virus can cross species to infect humans, few suspected cases of human-to-human transmission have been reported (*27*). However, the virus could acquire characteristics that allow it to be readily transmitted among humans, which could cause a worldwide influenza pandemic, with the potential for killing millions of people. In 1918, a pandemic of the "Spanish Flu" killed 20–50 million people worldwide (*26**,**27*).

Recently, the NIH Influenza Genomics Project was initiated; it will conduct rapid sequencing of the complete genomes of the several thousand known avian and human influenza viruses as well as those that emerge in the future. Approximately 60 genomes are expected to be sequenced each month. This project should also illuminate the molecular basis of how new strains of influenza virus emerge and provide information on characteristics that contribute to increased virulence. Many researchers believe that the H5N1 virus shows the greatest potential for evolving into the next human pandemic strain. Avian H9N2 viruses also have infected humans and have the potential to cause a pandemic. To prepare for this possibility, the development of vaccines to prevent infection with H5N1 and H9N2 viral strains is being supported (*28*). Researchers also are working to develop a live-attenuated vaccine candidate directed against each of the 15 hemagglutinin proteins that have been isolated, an effort that may speed the development of a vaccine against a potential pandemic strain.

Using reverse genetics, researchers developed a genetically engineered vaccine candidate (called a reference virus) against H5N1 in a matter of weeks, demonstrating the power of this technology (*29*). The new H5N1 candidate was tested in animals to confirm that it was no longer highly pathogenic (*29*), and vaccine manufacturers are using the reference virus to develop inactivated vaccines that will be evaluated in phase 1 and 2 clinical trials. Reverse genetics also has been used to identify a specific genetic mutation in a H5N1 viral gene, called PB2, which makes the virus especially lethal. This discovery may be useful in designing antiviral drugs and vaccine candidates (*30*).

Experiments also are being conducted in which genes isolated from the 1918 influenza strain are cloned into avirulent influenza strains. Researchers recently showed that the hemagglutinin gene from the 1918 virus conferred a high degree of pathogenicity to avirulent influenza strains when introduced into mice (*31*). These recombinant viruses and others are being evaluated in various animal models, including nonhuman primates, to further determine how genes of the 1918 virus contributed to its ability to spread so rapidly and cause so many deaths, and to understand the molecular basis for its unprecedented virulence. Previous research (*32*) established the foundation for developing a live-attenuated nasal flu vaccine that was approved by FDA in 2003 for use in healthy adults and children 5–49 years of age (*33*).

## West Nile Virus

West Nile virus (WNV), long endemic in Africa, West Asia, Europe, and the Middle East, represents a reemerging disease that only recently arrived in the United States. The virus first appeared in the New York City area in 1999, where WNV-related disease was reported in 62 persons. It has continued to spread throughout the United States in subsequent summers, infecting ever larger populations, particularly in 2003 (*34*). Research has led to several promising vaccine candidates against WNV (*35*). One of these, based on a licensed yellow fever vaccine virus that contains 2 WNV genes, has been tested in nonhuman primates; it is currently being evaluated in human clinical trials. A second vaccine developed at NIH uses an attenuated dengue virus into which WNV genes have been inserted. This vaccine protects monkeys and horses against WNV infection, and a clinical trial is now underway. Subunit and DNA vaccines against WNV are also in various stages of development and testing.

Several innovative therapies also are being tested to treat persons already infected with WNV. In a clinical trial at >60 sites across the United States and Canada, the protective effect of an immunoglobulin product is being tested in hospitalized patients who are at high risk for or who have WNV encephalitis (*36*). Technology also has been developed to screen large numbers of chemical compounds for antiviral activity. As of February 2005, 1,500 compounds had been screened in vitro, and 2% were shown to have antiviral activity against WNV. These compounds are undergoing further evaluation in hamster and mouse models of disease. Partnerships with small biotechnology companies have been formed to develop more sensitive and rapid tests for detecting WNV infections. Other studies are ongoing to evaluate the roles of various mosquito vectors and animal reservoirs in virus transmission, to test novel mosquito control methods, and to limit the impact of insecticide resistance on mosquito control (*37*).

## SARS

The emergence of SARS in Asia in late 2002, and the speed with which it was characterized and contained, underscores the importance of cooperation between researchers and public health officials (*38*). NIAID is focusing its resources on developing diagnostics, vaccines, and novel antiviral compounds to combat SARS-CoV. Basic research on the pathogenesis of the disease to identify appropriate targets for therapeutics and vaccines, as well as clinical studies to test new therapies, is also being supported. Among many projects that have received support are the development of a "SARS chip," a DNA microarray to rapidly identify SARS sequence variants, and a SARS diagnostic test based on polymerase chain reaction technology (*38*).

Researchers have developed 2 candidate vaccines, based on the SARS-CoV spike protein, that protect mice against SARS (*39**,**40*). Another promising vaccine protects against infection in monkeys when delivered intranasally (*41*). Passive immunization as a treatment for SARS patients is also being investigated. Both mouse and human antibodies against SARS can prevent infection when introduced into uninfected mice (*42**,**43*), and an international collaboration has developed a rapid method of producing human anti-SARS antibodies (*43*). In 2004, in vitro screening of >20,000 chemicals identified ≈1,500 compounds with activity against SARS-CoV, at least 1 of which has been selected by industry as a candidate for further clinical development (*38*).

## Potential Bioterror Agents

The September 11, 2001, attacks on the World Trade Center and Pentagon, and the subsequent anthrax attacks that infected 22 people and killed 5, propelled the U.S. government to expand its biodefense research program (*44*). These studies are based on 3 approaches: basic research aimed at understanding structure, biology, and mechanisms by which potential bioweapons cause disease; studies to elucidate how the human immune system responds to these dangerous pathogens; and development of the technology to translate these basic studies into safe and effective countermeasures to detect, prevent, and treat diseases caused by such pathogens (*44*).

At least 60 major NIAID initiatives involving intramural and extramural scientists and industrial partners were funded in fiscal years 2002–2004. Among them are funding for 8 Regional Centers of Excellence for Biodefense and Emerging Infectious Diseases Research and construction of 2 National Biocontainment Laboratories and 9 Regional Biocontainment Laboratories. These facilities will provide the secure space needed to carry out the nation's expanded biodefense research program (*44*).

The genomes of all biological agents considered to pose the most severe threats have been sequenced by researchers (*45*). In addition, programs have been expanded and contracts awarded to screen new chemical compounds as possible treatments for bioterror attacks. New animal models have been developed to test promising drugs, and repositories have been established to catalog reagents and specimens (*44*).

In addition, research to understand the body's protective mechanisms against pathogens is being pursued. The Cooperative Centers for Translational Research on Human Immunology and Biodefense will focus on studies of the human immune response to potential agents of bioterror, while other programs are focused on the innate immune system and the development of ways to boost innate immunity (*44*).

NIAID also has been very active in vaccine development as a biodefense countermeasure (*44*). The Institute has supported the development of a next-generation anthrax vaccine, known as recombinant protective antigen (rPA); it is undergoing clinical trials, and contracts for the Strategic National Stockpile to acquire it have recently been awarded. Several new smallpox vaccines also are being tested for safety and efficacy. Preliminary studies in mice and monkeys show that one of these, modified vaaccinia Ankara (MVA), protects against poxvirus infections (*46**,**47*). Clinical trials of the MVA vaccine are ongoing at NIAID Vaccine Research Center and elsewhere (*44*).

A clinical trial of a novel DNA vaccine against Ebola virus also is under way; human testing of an adenovirus-vectored Ebola vaccine is planned for 2005 (*48*). Vaccine manufacturing and clinical trials also are planned for a new, recombinant vaccine against plague that is highly effective in mice and nonhuman primates (*49*).

## Challenges for the Future

Scientists—government and academic, together with their industrial partners and international collaborators—have made great strides over the past 10 years in understanding many of the pathogenic mechanisms of emerging and reemerging infectious diseases. Many of these discoveries have been translated into novel diagnostics, antiviral and antimicrobial compounds, and vaccines, often with extraordinary speed.

However many challenges remain. Paramount among these is developing a safe and effective HIV vaccine. The evolution of pathogens with resistance to antibacterial and antiviral agents continues to challenge us to better understand the mechanisms of drug resistance and to devise new ways to circumvent the problem. These efforts will pave the way for developing countermeasures against deliberately engineered microbes.

If history is our guide, we can assume that the battle between the intellect and will of the human species and the extraordinary adaptability of microbes will be never-ending. To successfully fight our microbial foes, we must continue to vigorously pursue research on the basic mechanisms that underlie microbial pathogenesis and develop novel strategies to outwit theses ingenious opponents. The past 10 years have been challenging but no more so than will be the future.
